# BAY11 enhances OCT4 synthetic mRNA expression in adult human skin cells

**DOI:** 10.1186/scrt163

**Published:** 2013-02-06

**Authors:** Jason P Awe, Agustin Vega Crespo, You Li, Megerditch Kiledjian, James A Byrne

**Affiliations:** 1 Department of Molecular and Medical Pharmacology, University of California, Los Angeles, 650 Charles E. Young Drive South, 23-120 Center for Health Sciences, Los Angeles, CA 90095, USA; 2 Eli and Edythe Broad Center of Regenerative Medicine and Stem Cell Research, University of California, Los Angeles, 615 Charles E Young Drive South, Los Angeles, CA 90095, USA; 3 Department of Cell Biology & Neuroscience, Rutgers University, 604 Allison Road, Piscataway, NJ 08854, USA

## Abstract

**Introduction:**

The *OCT4 *transcription factor is involved in many cellular processes, including development, reprogramming, maintaining pluripotency and differentiation. Synthetic *OCT4 *mRNA was recently used (in conjunction with other reprogramming factors) to generate human induced pluripotent stem cells. Here, we discovered that BAY 11-7082 (BAY11), at least partially through an NF-κB-inhibition based mechanism, could significantly increase the expression of OCT4 following transfection of synthetic mRNA (synRNA) into adult human skin cells.

**Methods:**

We tested various chemical and molecular small molecules on their ability to suppress the innate immune response seen upon synthetic mRNA transfection. Three molecules - B18R, BX795, and BAY11 - were used in immunocytochemical and proliferation-based assays. We also utilized global transcriptional meta-analysis coupled with quantitative PCR to identify relative gene expression downstream of OCT4.

**Results:**

We found that human skin cells cultured in the presence of BAY11 resulted in reproducible increased expression of OCT4 that did not inhibit normal cell proliferation. The increased levels of OCT4 resulted in significantly increased expression of genes downstream of OCT4, including the previously identified *SPP1, DUSP4 *and *GADD45G*, suggesting the expressed OCT4 was functional. We also discovered a novel OCT4 putative downstream target gene *SLC16A9 *which demonstrated significantly increased expression following elevation of OCT4 levels.

**Conclusions:**

For the first time we have shown that small molecule-based stabilization of synthetic mRNA expression can be achieved with use of BAY11. This small molecule-based inhibition of innate immune responses and subsequent robust expression of transfected synthetic mRNAs may have multiple applications for future cell-based research and therapeutics.

## Introduction

Early embryonic development creates an inner cell mass in the developing embryo that, after delamination into the epiblast, initially lends itself exclusively to pluripotent stem cells capable of differentiating into any of over 200 cell types of the human body. The gene expression and transcriptional network that are expressed and regulated are well characterized [1-4]. One of the key pluripotency factors, *OCT4*, a Pou class 5 homeobox 1 transcription factor known as POU5F1, is expressed in human embryonic stem cells (hESCs), induced pluripotent stem cells, early epiblast, and germ cells, including primordial germ cells [5,6]. This transcription factor has been implicated in key pluripotency maintenance functions in both early embryogenesis, including acting as a master regulator in segmentation morphology and organogenesis via activation of key downstream signaling pathways, and activating tissue-specific transcription factors [7]. Interestingly, it has been shown that precise levels of OCT4 are needed during development, as repression leads to loss of pluripotency and subsequent trophectoderm differentiation and overexpression lead to differentiation into primitive endoderm and mesoderm, respectively [8]. It is clear that OCT4 plays a critical function in human developmental biology, and its role has been well defined in that it associates with other pluripotency factors, *SOX2 *and *NANOG*, whose mechanism to maintain a pluripotent phenotype involves upregulation and downregulation of over 4,600 genes through a protein network of these three proteins [9-11]. Thus, the delivery and stable expression of synthetic *OCT4 *mRNA and other synthetic mRNAs (synRNAs) may have multiple applications for future cell-based research and therapeutics.

The ability to reprogram easily obtainable human cells, such as skin cells, back into a pluripotent epigenetic state provides exciting new possibilities for *in vitro *research and patient-specific cellular therapeutics to regenerate our bodies following injury, disease, and age-based tissue degeneration [12]. However, the most promising method for reprogramming human somatic cells back into a pluripotent state - referred to as induced pluripotent stem cells - uses viruses to deliver the reprogramming factors (*OCT4, SOX2 *combined with *KLF4 *and *cMYC *or with *NANOG *and *LIN28*) into human somatic cells [13,14]. As these viruses randomly integrate into the genome, insertional mutagenesis is an important safety concern [15-17]. Alternatives to integrating DNA virus-based reprogramming include the use of episomal plasmids [18] and minicircles [19], protein-based reprogramming [20], and Sendai virus-based reprogramming [21]. Both of the episomal DNA-based reprogramming methodologies, however, still entail some risk of genomic recombination or insertional mutagenesis. The recombinant proteins used in protein-based reprogramming are challenging to generate and purify in the quantities required, and the RNA-based Sendai virus requires an extended period of culture in order to dilute out the viral particles [22]. Perhaps the most promising current integration-free reprogramming methodology for future patient-specific cellular therapeutics involves the direct transfection of RNAs into somatic cells (that is, synthetic whole mRNAs [23] or microRNAs [24] or both). SynRNAs encoding for five of the reprogramming factors (*OCT4, SOX2, KLF4, cMYC*, and *LIN28*) have been shown to reprogram human somatic cells back into a pluripotent state [23]. The most important of these delivered reprogramming factors is *OCT4*, as recent research has demonstrated that *OCT4*, in combination with certain small molecules, can itself induce a somatic cell to reprogram to pluripotency without requiring assistance from the other factors [25].

Here, we examined the expression of synthetic *OCT4 *mRNA following transfection into adult human skin cells, investigated whether various small molecules (B18R, BX795, and BAY11) could significantly increase synthetic *OCT4 *mRNA expression, and used transcriptional analysis of OCT4 downstream genes to determine whether the OCT4 protein maintained its functionality as a transcription factor.

## Materials and methods

### Ethics statement

Written approval for human skin biopsy procedures and human fibroblast derivation, culture, and experimental use was obtained from the Stanford University Institutional Review Board, the Stanford University Stem Cell Research Oversight (SCRO) committee, and written informed consent was obtained from each individual participant. Biopsy material used in this study was obtained and initially analyzed at Stanford University, as previously described [26], and transferred to the University of California at Los Angeles (UCLA) through a material transfer agreement. Written approvals for the experiments performed in this study were obtained from the UCLA Institute Biosafety Committee and the UCLA SCRO committee.

### *In vitro *culture of primary human skin cells

The human skin-derived (HUF1) primary cell line used in this study was obtained from a 4-mm adult skin punch biopsy and cultured as described [26]. Briefly, all human biopsy-derived cells were cultured in complete DMEM/F-12 media consisting of Dulbecco's modified Eagle's medium nutrient mixture F-12 (DMEM/F-12) supplemented with 10% fetal bovine serum (FBS), 1 × minimum essential medium (MEM) non-essential amino acids, 1 × Glutamax, and 100 IU/mL penicillin-streptomycin (all from Invitrogen Corporation/Gibco, Grand Island, NY, USA) and maintained at 37°C in a 5% CO_2_ incubator. Culture media were changed every two days. Cells were allowed to expand to 80% to 90% confluency before passaging with 0.05% trypsin-EDTA (Invitrogen Corporation) and replating at a 1:3 ratio. A large bank of early-passage HUF1 cells was cryopreserved in culture media supplemented with 10% dimethyl sulphoxide (DMSO) (Sigma-Aldrich, St. Louis, MO, USA). All research adhered to National Academy of Sciences guidelines.

### *In vitro *culture of H9 human embryonic stem cells

H9 hESCs (UCLA Broad Stem Cell Research Center-Stem Cell Core) were cultured in standard ESC conditions as published [26]. Briefly, hESCs were cultured in medium consisting of DMEM/F-12 supplemented with 20% knockout serum replacement, 1 × Glutamax, 1 × non-essential amino acids, 100 IU/mL penicillin-streptomycin (all from Invitrogen Corporation), 1 × β-mercaptoethanol (Millipore Corporation, Billerica, MA, USA), and 10 ng/mL recombinant human basic fibroblast growth factor (Globalstem, Rockville, MD, USA) and maintained at 37°C in a 5% CO_2_ incubator. Media were changed daily.

### Cell proliferation analysis

**Figure 1:**
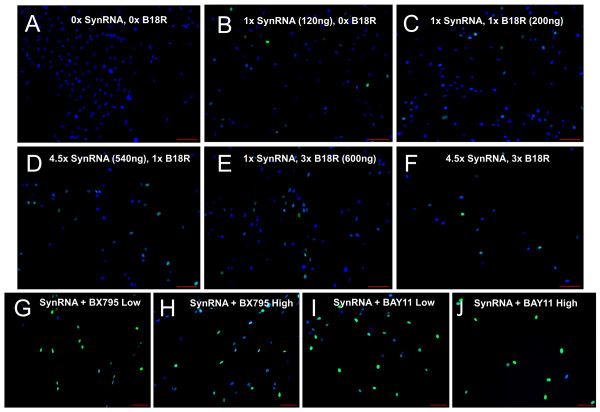
**Immunocytochemical analysis of OCT4 expression**. **(a-j) **Adult human dermal fibroblasts (HUF1) were exposed to different synthetic mRNA (synRNA) concentrations to stabilize and promote homogenous OCT4 expression. Addition of 1 × or 4.5 × synRNA did not yield significantly higher OCT4 expression (b,d). Addition of B18R was added to assuage interferon (IFN) signaling, although 1 × and 3 × concentrations did not yield significant increases in OCT4 expression (c,e). 4.5 × mRNA at 520 ng and 3 × B18R at 600 ng/mL also did not yield significant stabilization of OCT4 (f). Small-molecule compounds of BX795 at low (0.001 µM) and high (1 µM) concentrations did yield robust expression of OCT4 (g and h, respectively). BAY11 at low (0.01 µM) and high (1 µM) concentrations also stabilized OCT4 to an even greater degree than BX795 (i and j, respectively). Scale bar represents 100 µM. DAPI (4′,6-diamidino-2-phenylindole) staining is represented in blue; OCT4 staining is represented in green. BAY11, BAY 11-7082.

BAY11 and BX795 were purchased from InvivoGen (San Diego, CA, USA) (catalog code tlrl-b82 and tlrl-bx7). Both inhibitors were diluted initially in DMSO to a stock concentration of 100 mM and stored in aliquots at −20°C. All further dilutions to working concentrations were carried out in filter-sterilized Millipore water. Fresh aliquots were used for each daily transfection. B18R recombinant protein was purchased from eBioscience (San Diego, CA, USA), aliquoted, and stored at −80°C. HUF1 cells were grown as described alone in complete DMEM/F-12 media. For cell proliferation experiments, HUF1 cells were plated onto gelatin-coated six-well plates (Sigma-Aldrich) at a concentration of 50,000 cells (based on a doubling time of 34 hours) and left to sit for 24 hours to adhere in the presence of BAY11 or BX795 (as indicated in Figure 1), and this was considered day 0. Every day at the 24-hour mark, media were changed with fresh drug and B18R (when applicable). On day 5, cells were washed twice with 1 × phosphate-buffered saline (PBS) (Invitrogen Corporation) and cells detached with 1 mL per well of a six-well plate via 0.05% trypsin-EDTA for 5 minutes at 37°C in a 5% CO_2_ incubator. Cells were then quenched with 4 mL of standard fibroblast culture media, centrifuged at 120*g *for 5 minutes, and resuspended in 1 mL of culture media along with Trypan blue stain (Invitrogen Corporation) for cell counting by using a standard hemocytometer. All cell counts were performed in quadruplicate.

### Synthetic mRNA dilutions

For the initial 'mRNA pooling' experiments, synRNA of each of the five reprogramming factors was generated in-house, aliquoted, and stored at −80°C as previously described by Warren and colleagues [23]. Briefly, in accordance with the approach of Warren and colleagues, a 170:160:420:130:120 stoichiometric ratio corresponding to 'KMOSL' factors (which consisted of *KLF4*:*c-MYC*:*OCT4*:*SOX2*:*LIN28*, respectively) was used for the initial 'pooling' experiments. SynRNA stock concentrations for KMOSL were 1,200, 750, 1,500, 650, and 600 ng/µL, respectively, and were all diluted to 100 ng/µL stocks with Tris-EDTA pH 7.0. To pool these together with the same stoichiometric ratios used by Warren and colleagues, 121.4, 114.3, 300, 92.9, and 64.3 µL, respectively, were added together on ice and under sterile conditions, mixed, and immediately placed into 12-µL aliquots for storage at −80°C. Thus, each vial had a cocktail of the five factors at 100 ng/µL to yield 1,200 ng total per aliquot of synRNA, and the synRNA cocktails - except for *OCT4*, which was present at a 3 × molar concentration - were formulated to yield equal molarity. For the later *OCT4 *mRNA experiments, only *OCT4 *synRNA, obtained from a commercial vendor (Stemgent, San Diego, CA, USA), was used.

### Synthetic mRNA transfection

All work was carried out in strict RNAse-free conditions. SynRNA was thawed on ice and quickly diluted before degradation. SynRNA was diluted 5 × by using Opti-MEM basal media (Invitrogen Corporation) with 12 µL of synRNA at 100 ng/µL placed into 48 µL of Opti-MEM. RNAiMAX was used at 5 µL per microgram of RNA - 1.2 µg of total synRNA and thus 6 µL of RNAiMAX - and was diluted 10 × by diluting 6 µL of RNAiMAX into 54 µL of Opti-MEM. Each dilution was separate, and the tube was mixed. The two dilutions were pooled, mixed, and incubated at room temperature for 15 minutes. This mixture was added directly into fibroblast culture media without antibiotics, as required by protocol. Final culture volumes in each well were 500 µL. The cells were left to incubate with the synRNA for 4 hours, after which the media were replaced with fresh BAY11 or BX795 along with B18R all in standard fibroblast media. For the *OCT4 *mRNA single-factor assay, daily transfections were carried out as above onto 25,000 HUF1 cells plated into a 24-well plate by using synthetic *OCT4 *mRNA (Stemgent) over the course of 5 days, with or without the addition of BAY11. B18R was not included during the single-factor transfections.

### Immunocytochemistry

Cultured cells were fixed in 4% paraformaldehyde/1 × PBS for 15 minutes, washed twice with 1 × PBS supplemented with 100 mM glycine for 5 minutes, and incubated with permeabilization buffer consisting of 0.1% Triton X-100 (Sigma-Aldrich) in 1 × PBS for 30 minutes at room temperature. Blocking was performed with 4% goat serum in Blocker Casein in PBS (Thermo Scientific, Rockford, IL, USA) for 60 minutes at room temperature. Then OCT4 (C-10) mouse anti-human monoclonal IgG_2b_ 1:200 (Santa Cruz Biotechnology, Inc., Santa Cruz, CA, USA; catalog code sc-5279) was added to 4% goat serum in Casein-PBS and incubated overnight at 4°C. The next day, cells were washed three times with 1 × PBS before Alexa Fluor 488 goat anti-mouse IgG 1:200 (Invitrogen Corporation) was added to 4% goat serum in Casein-PBS and incubated for 1 hour at room temperature. The cells were rinsed and stained with SlowFade Gold antifade reagent with 4′,6-diamidino-2-phenylindole (DAPI) (Invitrogen Corporation) for 10 minutes, followed by two 1 × PBS washes. Cultures were visualized with an AxioCam MR Monocolor Camera by using AxioVision Digital Image Processing Software (Axio Observer Inverted Microscope; Carl Zeiss, Jena, Germany). Three images per well were used for luminosity cell counts evaluated with Adobe Photoshop (Adobe Systems, Mountain View, CA, USA). In each replicate, 10 cells located in the top left frame of the picture were characterized with the luminosity tool in Photoshop.

### OCT4 quantification

The OCT4 stabilization assay was carried out via a 10 × dilution of the 100 ng/µL synRNA stock across 10 wells for a total of 120 ng per well in a 24-well plate. In total, 10,000 cells per well were plated, and all synRNA dilutions were carried out as stated above. Cells were cultured in standard fibroblast media, without antibiotics, overnight. The day after the initial plating, each combination of BAY11 or BX795, with or without B18R, was diluted directly into cell culture medium for 24 hours to precondition the medium. SynRNA was then transfected in every 24 hours with fresh replacement of media and drug for 5 days. OCT4 was then quantified via immunocytochemical analysis and analyzed as previously mentioned via immunocytochemical and luminosity measurements in Photoshop.

### Global transcriptional meta-analysis

In total, 25,000 passage 5 HUF cells were plated into individual wells in a 24-well plate in standard DMEM/FBS media without antibiotics. Twelve hours later, the cells were incubated with 1 µM BAY11 + 200 ng/mL B18R over two wells, whereas the other two wells received just B18R at 200 ng/mL final concentration. The final volume was 500 µL in each well. After 24 hours of incubation, the cells were transfected with the modified synRNA as detailed above. This was carried out every day for 3 days, and after the last incubation period of 24 hours, the cells were harvested for mRNA by using a Roche High Pure RNA Isolation Kit in accordance with the instructions of the manufacturer (Roche, Indianapolis, IN, USA). hESCs and control fibroblasts were harvested in the same manner. Microarray analysis was carried out as published [27]. Briefly, total RNA was used for an Affymetrix Differential Gene Expression Assay Human Genome U133 Plus 2.0 Array (Genoseq UCLA) for global transcriptional analysis by using standard Affymetrix protocols (Affymetrix GeneChip Expression Analysis Technical Manual, rev. 3. 2001). Uploading and cluster analysis of the CEL files between replicate samples were carried out through GeneSifter (VizX Labs, Seattle, WA, USA[28]) by using the Advanced Upload Method and were normalized by using the Affymetrix Microarray Analysis Suite (MAS) 5.0 algorithm. The following CEL files were used for this analysis: HUF1 cells transfected with synRNA but not treated with BAY11 (GSM994323 and GSM994324), HUF1 cells transfected with synRNA and treated with BAY11 (GSM994325 and GSM994326), HUF1 cells not transfected with synRNA and not treated with BAY11 (GSM994327 and GSM994328)

and H9 human embryonic stem cells (GSM994321 and GSM994322). Data from control HUF cells were used as a baseline control to compare the replicates of HUF cells with or without BAY11 and hESCs. All experimental details for the microarray analysis, including all original CEL files, have been made publically available at the Gene Expression Omnibus [29] GSE40444.

### Quantitative polymerase chain reaction analysis

Total mRNA that was harvested from the global transcriptional meta-analysis was also used in reverse transcription-polymerase chain reaction (RT-PCR) for quantitative PCR analysis. The RNA yield and quality were determined by using the GE NanoVue Spectrophotometer (GE Healthcare Life Sciences, Piscataway, NJ, USA). For hESCs and for all HUF lines, 1 µg and 180 ng of RNA, respectively, were reverse-transcribed by using the Transcriptor First Strand cDNA Synthesis Kit (Roche) and anchored-oligo(dT)_18_ and random hexamer primers. Quantitative RT-PCR (QPCR) relative expression experiments used a LightCycler 480 Real-Time PCR System (Roche), and data were further analyzed with LightCycler 480 Software release 1.5.0. Primers and probes were designed and ordered from Roche's Universal ProbeLibrary. Primers for the genes are listed as follows: Homo sapiens secreted phosphoprotein 1 (*SPP1*), transcript variant 2, mRNA (NM_000582.2) forward primer: cgcagacctgacatccagt, reverse primer: ggctgtcccaatcagaagg, probe #61; Homo sapiens solute carrier family 16, member 9 (monocarboxylic acid transporter 9) (*SLC16A9*), mRNA, (NM_194298.2), forward primer: gatgcctttggtgaaggaaa, reverse primer: cacagagactgcagacaggact, probe #64; Homo sapiens growth arrest and DNA-damage-inducible, gamma (*GADD45G*), mRNA, (NM_006705.3), forward primer: cagccaaagtcttgaacgtg, reverse primer: cctggatcagcgtaaaatgg, probe #71; *DUSP4*-001 dual-specificity protein phosphatase 4 (ENST00000240100.2), forward primer: tgcatcccagtggaagataac, reverse primer: gcagtccttcacggcat, probe #17; Homo sapiens hypoxanthine phosphoribosyltransferase 1 (*HPRT1*), mRNA, (NM_000194.2), forward primer: tgaccttgatttattttgcatacc, reverse primer: cgagcaagacgttcagtcct, probe #73; *GAPDH*-001 glyceraldehyde-3-phosphate dehydrogenase (ENST00000229239.5), forward primer: gctctctgctcctcctgttc, reverse primer: acgaccaaatccgttgactc, probe #60. cDNA (5 ng) that was reverse-transcribed in the RT-PCR per sample was used in a 20-µL reaction that consisted of 10 µM UPL probe, 2 × LightCycler 480 Probes Master, and 20 µM forward and reverse primers. Duplicate experimental samples were paired by using the all-to-mean pairing rule with two housekeeping genes run in duplicate for advanced relative quantification.

### Statistical analysis

Results are presented as mean ± standard deviation. The statistical significance of differences for cell proliferation analysis, immunocytochemical luminosity-based quantification, and QPCR results were evaluated by using Statistical Package for the Social Sciences (SPSS) 20 (IBM Corporation, Chicago, IL, USA). Analysis of variance, *t *test for independent samples, and Mann-Whitney *U *test were considered statistically significant at a *P *value of less than 0.05.

## Results

### Immunocytochemical detection of OCT4 demonstrating robust stabilization

**Figure 2:**
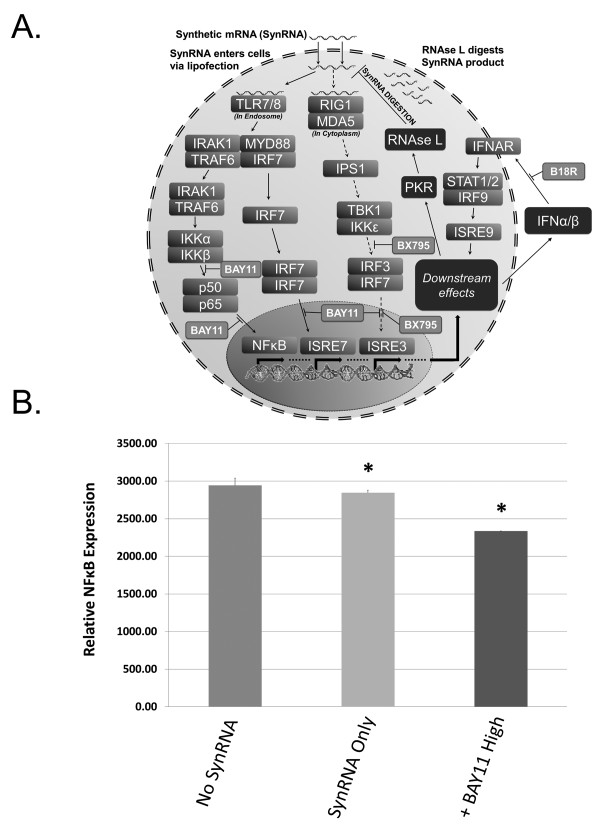
**Overview of molecular signaling pathway and quantitative real-time polymerase chain reaction analysis of nuclear factor-kappa-B (NF-κB)**. **(a) **Dotted line represents the molecular pathway that BX795 inhibits, specifically at IKKε. The pathway with non-dotted lines represents the additional pathways that BAY11 inhibits, including the IKKβ and IRF7 pathways. **(b) **Relative NF-κB levels with and without BAY11. Overall levels decrease in the presence of BAY11, contributing to the increase in OCT4 expression previously seen. Asterisks indicate statistically significant changes in expression where *P *value was less than 0.05. BAY11, BAY 11-7082; IFNAR, interferon-α/β receptor; IKK, IκB kinase; IRAK1, interleukin-1R-associated kinase 1; IRF, interferon regulatory factor; ; ISRE, interferon stimulated response element; MDA5, melanoma differentiation-associated gene 5; MYD88, myeloid differentiation primary-response gene 88; RIG-I, retinoic acid-inducible gene I; STAT1/2, signal transducer and activator of transcription 1/2; TLR7/8; TRAF6, tumor necrosis factor receptor-associated factor 6.

**Figure 3:**
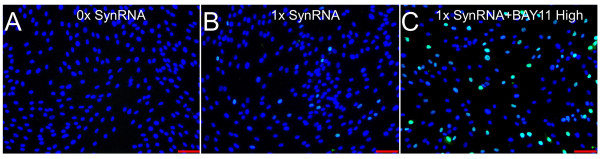
**Immunocytochemical analysis of OCT4 expression with *OCT4 *mRNA alone**. **(a-c) **HUF1 cells were exposed to 0 × and 1 × synthetic *OCT4 *mRNA without B18R and in the presence or absence of BAY11. The addition of BAY11 was noted to again stabilize and allow homogenous expression of OCT4 without the presence of B18R or any other mRNAs in culture. Scale bar represents 100 µM. DAPI (4′,6-diamidino-2-phenylindole) staining is represented in blue; OCT4 staining is represented in green. BAY11, BAY 11-7082; synRNA, synthetic mRNA.

In our initial attempts to repeat the reprogramming methodology of Warren and colleagues [23] for transfecting synthetic (syn) *OCT4 *mRNA into human adult fibroblasts, we observed significant degradation (defined here by low-level and heterogeneous OCT4 expression) of the *OCT4 *synRNA. Interestingly, this degradation appeared to be specific to *OCT4 *and did not affect *SOX2, KL4, cMYC*, and *LIN28 *(data not shown), but the reasons for this are not yet clear. No detectable OCT4 protein was observed in the untransfected human adult fibroblasts (HUF1) (Figure 1a). After transfection of 120 ng of synRNA (as reported by Warren and colleagues [23]), we saw broad heterogeneity and very low expression of OCT4 upon immunocytochemical analysis (Figure 1b). Secondly, addition of Vaccinia virus decoy receptor for type I interferons (IFNs), identified as B18R, resulted in no statistically significant increase in OCT4 expression (Figure 1c), and no significant increase was observed following the use of higher amounts of synRNA (Figure 1d), B18R (Figure 1e), or both (Figure 1f). These results demonstrate that increasing synRNA concentration and mitigating IFN signaling via B18R are not sufficient to permit robust OCT4 expression from synRNA.Next, we analyzed the immune response pathways to identify alternative potential small-molecule candidates that could potentially block the intra-cellular immune response pathway and thus stabilize OCT4 expression from synRNA (Figure 2a). The degradation of synthetic single-stranded RNA (ssRNA) is elicited through two distinct pathways (Figure 2a). First, interaction between pathogen-associated molecular patterns, including ssRNA [30], leads to activation of the mitogen-activated protein kinases (MAPKs) and the IκB kinase (IKKα and IKKβ), which subsequently turn on nuclear factor-kappa-B (NF-κB), via phosphorylating IκBα [31]. The second pathway uses another subset of pattern recognition receptors - endosomal Toll-like receptors [32], melanoma differentiation-associated gene 5 (MDA-5), and retinoic acid-inducible gene I (RIG-1) - that activate a distinct pathway which requires IKK-related kinases, IKKε, and TANK-binding kinase 1 (TBK_1_) and which also leads to activation of NF-κB and NF-κB-based gene transcription [31]. Both pathways recognize foreign RNA, and subsequent NF-κB-based activation of IFN regulatory factor (IRF) leads to type I IFN production [33-35]. Thus, inhibitor κB (IκB) proteins, normally sequestering NF-κB in the cytoplasm, must undergo phosphorylation via IκB kinase α (IKK) and subsequent rapid proteasome degradation, allowing NF-κB transcriptionally based regulated IFN production [36]. BAY11 inhibits IκBα phosphorylation (specifically, IRF7 production) [37,38], whereas BX795 inhibits TBK_1_ and IKKε. Both BAY11 and BX795 ultimately may inhibit many degradation-associated cytokines, RNAse L, and chemokines [31] (Figure 2a). Warren and colleagues found that use of modified ribonucleoside bases and a phosphatase treatment to reduce the signaling through RIG-1, coupled with an IFN inhibitor B18R, led to significantly reduced innate immune responses [39], although it did not completely eliminate this synRNA degradation in our experiments. We then asked whether two other candidate small molecules (BX795 and BAY11) might efficiently block the intracellular immune response to synthetic RNA and stabilize OCT4 expression (Figure 2a). When combined with 120 ng of total synRNA, BX795 at the low concentration (0.001 µM) induced broad stabilization and homogenous expression of OCT4 (Figure 1g). We then investigated whether increasing the concentration of BX795 to 1 µM would increase OCT4 expression. As shown in Figure 1h, a greater number of cells did express OCT4 but at a level comparable to that of BX795 at 0.001 µM (Figure 1g). Next, we tested a similar compound, BAY11, at a low concentration (0.01 µM) and obtained robust OCT4 stabilization and more uniform expression (Figure 1i). This was followed by one last condition tested with BAY11 at a high concentration (1 µM), and we found dramatically increased OCT4 expression (Figure 1j). In comparison, the original conditions of just synRNA and B18R yielded low and very heterogeneous expression of OCT4 (Figure 1b,c). To further investigate the reproducibility of this phenomenon, we used *OCT4 *synRNA alone, bought from an independent commercial manufacturer (Stemgent), and were able to duplicate the response seen in Figure 1, even in the absence of B18R (Figure 3a-c). This indicates that BAY11, and not the modified nucleobases or B18R, is the main contributing factor that is allowing the mRNA stabilization. In an effort to investigate whether the NF-κB-based innate immune response pathway is involved in this *OCT4 *stabilization phenomenon (as we hypothesized), we performed an assay without transfection of B18R or multiple pooled mRNAs. Not only did we still observe the stabilization of OCT4 expression but we also observed, through quantitative PCR, a statistically significant decrease in NF-κB expression when in the presence of BAY11 after daily transfection of synthetic *OCT4 *mRNA alone (Figure 2b). This correlative evidence suggests that the reproducible and robust increase in *OCT4 *mRNA expression we have observed is due, at least in part, to the inhibition of the NF-κB-based innate immune response pathway.

### Quantitation of immunocytochemistry and cell proliferation analysis

**Figure 4:**
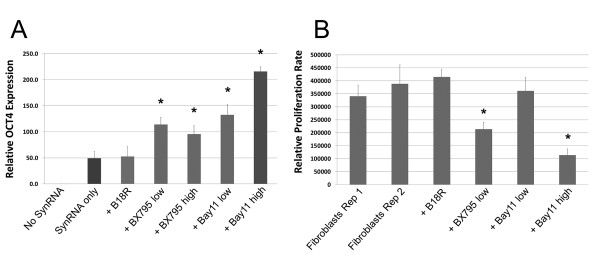
**Relative OCT4 expression levels and relative proliferation rate**. **(a) **Quantitative analysis of immunocytochemically detected OCT4 expression. **(b) **Proliferation analysis following exposure to small molecules. Asterisks indicate statistically significant increases or decreases over controls where *P *value was less than 0.05. BAY11, BAY 11-7082; synRNA, synthetic mRNA.

Luminosity-based measurements were used to quantify which conditions yielded significantly increased relative expression of OCT4. Fluorescent imaging demonstrated that BAY11 at 1 µM yielded the highest statistically significant relative expression of OCT4, followed closely by BAY11 at 0.01 µM (Figure 4a). There were no differences between the expression levels of OCT4 induced by BX795, and both were lower than the BAY11 concentrations (Figure 4a). We then conducted a cell proliferation assay on the HUF cells and found that both concentrations of BX795 and BAY11 at 1 µM have significant cell proliferative defects when compared with controls. However, BAY11 at 0.01 µM had the best compromise between robust OCT4 expression and no statistically significant reduction in cell proliferation compared with controls (Figure 4b). According to these findings, BAY11 used at 0.01 µM yielded optimal OCT4 expression without cell proliferative defects.

### Global transcriptional meta-analysis and quantitative polymerase chain reaction

**Figure 5:**
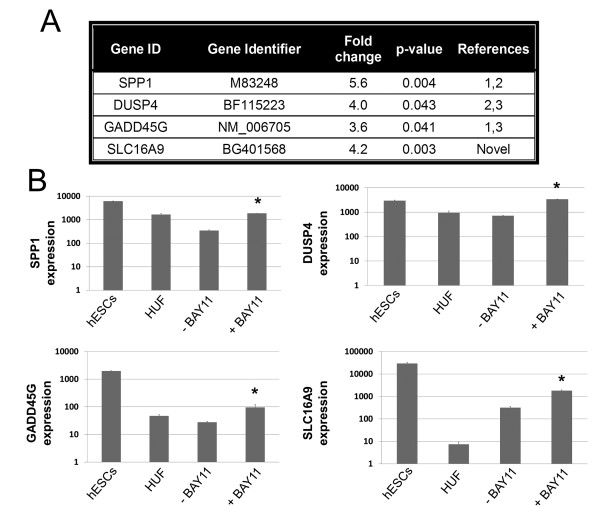
**Microarray analysis and quantitative real-time polymerase chain reaction (QPCR) of genes upregulated by BAY11**. **(a) **Microarray data show four putative gene targets that were identified by over 3 × fold changes in human fibroblast (HUF) cells treated with BAY11compared with untreated HUF cells over the course of 3 days of daily synthetic mRNA transfections. **(b) **QPCR analysis confirmed microarray results for the four genes and demonstrated that relative expression of the four genes becomes more similar to human embryonic stem cell (hESC) expression in the presence of BAY11. Asterisks indicate statistically significant increases over control cells not receiving BAY11 where *P *value was less than 0.05. BAY11, BAY 11-7082; *DUSP4*, dual-specificity phosphatase 4; *SLC16A9*, solute carrier family 16, member 9.

Next, we investigated whether global transcriptional meta-analysis could ascertain any early easily activated downstream transcriptional targets of OCT4 that may also be upregulated because of *OCT4 *stabilization in the presence of BAY11. Pairwise analysis of control and BAY11-treated fibroblasts revealed a number of probe sets upregulated (*P *<0.05, fold change >3). The differentially upregulated genes (when compared with our baseline HUF cells), in the presence of BAY11, were then cross-referenced with these genes in the ESC microarray. Further comparison of the downstream targets of OCT4 was carried out with previously reported results from the chromatin immunoprecipitation paired-end ditags methodology, global expression profiling, and chromatin-immunoprecipitation analysis [9-11]. From these data, four putative gene targets were found to be consistently upregulated: *SPP1, DUSP4, GADD45G*, and *SLC16A9 *(Figure 5a). These putative genes were confirmed with QPCR; however, the differential levels of gene expression observed via QPCR were, for unknown reasons, generally lower than those observed for microarray analysis. We demonstrated that, in the presence of BAY11, the expression of these four OCT4 early target genes was significantly increased relative to untreated HUF cells (Figure 5b). Interestingly, in the presence of BAY11, HUF cell expression of these four genes more similarly matched expression levels of hESCs, suggesting that BAY11 could be useful in reprogramming by upregulating key pluripotency-associated genes. Additionally, while *SPP1, DUSP4*, and *GADD45G *were previously found to be bound and regulated by OCT4 [9-11], we found a putative novel OCT4 target gene, *SLC16A9*, that has been previously shown through microarray analysis to also be upregulated in ESCs. Thus, through microarray analysis and QPCR, we detected and confirmed four early gene targets that are upregulated in the presence of BAY11, indicative of functional OCT4 expression.

## Discussion

In this study, we provide the first significant and reproducible evidence, collected over four independent experiments, in support of the hypothesis that BAY11 can significantly increase the expression of OCT4 in human adult dermal fibroblasts from transfected synRNA without negatively impacting cell proliferation. We demonstrated that this response involves NF-κB-based innate immune responses and is independent of the modified nucleobases and addition of B18R. Also, we were able to reproduce the robust expression of OCT4 by using single-factor mRNA synthesized from an independent company (Stemgent) rather than our original source of synRNA (generated in-house). Importantly, OCT4 can significantly upregulate its putative early target downstream genes, including *SPP1, DUSP4*, and *GADD45G*. For example, dual-specificity phosphatase 4 (*DUSP4*) plays a role in cellular proliferation and differentiation via phosphatase activity in the MAPK pathways, and previous studies reported that deletion of this gene causes a significant decrease in the cell proliferation rate [40]. Similarly, *c-MYC *has many downstream targets that enhance cell proliferation [13], and *DUSP4 *may have similar functions in reprogramming to increase cell proliferation. We also used global transcriptional analysis to identify a novel OCT4 target gene (*SLC16A9*) and demonstrated that *SLC16A9 *was significantly upregulated following BAY11-based treatment of adult human skin cells transfected with synthetic *OCT4 *mRNA. *SLC16A9 *(or solute carrier family 16, member 9) is a monocarboxylic acid transporter, which is interesting as highly glycolytic cells commonly express monocarboxylate transporters [41]. Interestingly, *SLC16A9 *is part of the monocarboxylate transporter family of H^+^/lactate symporters capable of bidirectional transport of lactic acid across the plasma membrane [42]. This is an important finding as ESCs have been found to be primarily glycolytic and have very few mitochondria [43]. Thus, ESCs, and most malignant cancers, express this glycolytic phenotype, even in the presence of oxygen, an effect known as the 'Warburg effect', and must efflux lactate to prevent toxic intracellular buildup of lactate [44]. Therefore, upregulation of *SLC16A9 *could be another avenue by which to increase reprogramming efficiency not only in mRNA-based reprogramming but also in other reprogramming methodologies.

It is interesting to note that, out of the various reprogramming factors we analyzed (*OCT4, SOX2, KLF4, cMYC*, and *LIN28*), only *OCT4 *triggered an intracellular immune response that resulted in very low heterogeneous expression. Whether *OCT4 *more closely resembles an evolutionarily recognized single-stranded virus that human somatic cells have evolved defense mechanisms to is an interesting hypothesis but as of yet is unproven. Also, exactly how human fibroblasts recognize and degrade transfected human *OCT4 *RNA and whether the polyA or 3′ untranslated region sequences play a role are unclear. We propose that human cells use the same intracellular immune response pathways that degrade viral single-stranded mRNA to degrade single-stranded *OCT4 *mRNA. The difference in recognition and degradation between synthetic *OCT4 *and endogenous *OCT4 *may reside in the relatively long half-life of viral mRNA and synRNA compared with the relatively short half-life of endogenous *OCT4 *mRNA (which is nevertheless continuously transcribed when expressed). This is an interesting area for future study. It should also be noted that, while the immunocytochemical assays we have used in this study do not distinguish between endogenous and exogenous OCT4 protein, we do not consider that this is a significant concern or changes the conclusions of our paper, as no detectable OCT4 protein was observed via the immunocytochemical assay in the human dermal fibroblasts without *OCT4 *synRNA transfection. It is also interesting to note that, for unknown reasons, the RT-PCR primers we have previously used to detect endogenous *OCT4 *mRNA did not work with synthetic *OCT4 *mRNA. Therefore, we focused our quantitative analysis in this study on the increased amount of OCT4 protein generated in the synRNA transfected cells and the significant upregulation of genes downstream of OCT4. In regard to the detection of only four OCT4 putative targets, it is unclear to us why a larger number of OCT4 downstream genes did not get upregulated, although we propose that the four putative OCT4 targets we identified in this study may potentially represent some of the earliest or easiest genes to upregulate (or both), hence their detection in our microarray and QPCR assays, while other OCT4 targets may require additional factors or time (or both) to express to detectable levels. To date, we have observed that BAY11 can significantly and reproducibly stabilize *OCT4 *over a 3-day and 5-day culture. It will be interesting to observe whether BAY11 can maintain this stabilized OCT4 expression over longer periods of time without inducing any negative or toxic effects (or both) on the cells. This long-term synRNA stabilization is critical toward almost all future applications of BAY11.

This research represents the first step toward using small molecules to augment the expression of various synRNAs in adult human somatic cells. At present, the molecular mechanism involved in the increased presence of OCT4 protein is not clear and may involve increased mRNA stability, translation, or improved transfection efficiency. Given that BAY11 has been found to suppress IRF7 production [37,38], tempering the innate immune response-mediated decay of exogenous mRNA is a plausible mechanism and likely involves increased mRNA stability of the transfected *OCT4 *mRNA. This is an interesting area for future study. In addition, our results indicate that BAY11 may be an important adjuvant to augment reprogramming to pluripotency [23], differentiation to endoderm [8], and/or expression of other potentially useful synRNA-derived factors. One of the most promising aspects of this study is the potential that BAY11 may be useful in stabilizing synthetic *OCT4 *mRNA to facilitate the generation of patient-specific induced pluripotent stem cells. This is especially relevant given that past findings have detailed that perhaps one of the biggest roadblocks during reprogramming via frequent mRNA transfections is an innate immune response [45]. Therefore, for the first time, we show that a small molecule, BAY11, can mitigate this innate immune response and partially prevent degradation of transfected synRNA.

## Conclusions

We conclude that BAY 11-7082 (BAY11) can significantly increase the expression of OCT4 following transfection of synRNA into adult human skin cells. This small molecule-based stabilization of synRNA expression may have multiple applications for future cell-based research and therapeutics.

## Abbreviations

BAY11, BAY 11-7082; DMEM/F-12, Dulbecco's modified Eagle's medium/F-12; DMSO, dimethyl sulphoxide; *DUSP4*, dual-specificity phosphatase 4; EDTA, ethylenediaminetetraacetic acid; ESC, embryonic stem cell; FBS, fetal bovine serum; hESC, human embryonic stem cell; HUF1, human fibroblast; IκB, inhibitor κB; IFN, interferon; IKK, inhibitor κB kinase α; IRF, interferon regulatory factor; KMOSL, *KLF4*:*c-MYC*:*OCT4*:*SOX2*:*LIN28*; MAPK, mitogen-activated protein kinase; MEM, minimum essential medium; NF-κB, nuclear factor-kappa-B; PBS, phosphate-buffered saline; PCR, polymerase chain reaction; QPCR, quantitative real-time polymerase chain reaction; RIG-I, retinoic acid-inducible gene I; RT-PCR, reverse transcription-polymerase chain reaction; SCRO, Stem Cell Research Oversight; *SLC16A9*, solute carrier family 16, member 9; ssRNA, single-stranded RNA; synRNA, synthetic mRNA; TBK_1_, TANK-binding kinase 1; UCLA, University of California at Los Angeles.

## Competing interests

JAB receives research funding and is a scientific consultant for Fibrocell Science, Inc. (Exton, PA, USA) and has a patent on the work included in this article. AVC receives research funding for Fibrocell Science, Inc. JPA has a patent on the work included in this article. YL and MK declare that they have no competing interests.

## Authors' contributions

JAB helped to design experiments and write the manuscript. JPA helped to design experiments and write the manuscript and performed experiments. YL and MK generated synthetic mRNA. AVC assisted with cell culture. All authors read and approved the final manuscript.

## Authors' information

JAB is an assistant professor at UCLA. The long-term aims of his research are to create and use patient-specific induced pluripotent stem cell technology to treat diseases such as Parkinson's, heart disease, and diabetes and to promote recovery from spinal cord injuries and regenerate human organs, tissues, and cells as they age, mutate, and die.
